# Supplementary material for: Adoption of Sustainable Agriculture Practices through Participatory Research: A Case Study on Galapagos Islands Farmers Using Water-Saving Technologies
Source: Plants (Basel). 2022 Oct 26;11(21):2848. doi: 10.3390/plants11212848 (PMC9654590; doi:10.3390/plants11212848)
Supplement: Supplementary file 1 [file plants-11-02848-s001.zip › plants-1920729-supplementary.pdf]

plants MDPI

Table S1. Plant traits for each species organized by water-saving technologies and seasons. Average values, differences (expressed as percentage in italics) between treatments and seasons are displayed. Underlined values show that dry season and control treatments are higher than the other variable.

Island			Santa Cruz			Floreana		
Species	Trait	Season	Groasis Waterboxx	Control	*Treatment difference*	Groasis Waterboxx	Control	*Treatment difference*
Capsicum annuum	Plant productivity (kg)	Wet	1.039	0.931	*5.47%*	-	-	-
		Dry	1.873	3.613	*-31.71%*	-	-	-
		Season difference	*-28.66%*	*-59.02%*		-	-	-
	Fruit number	Wet	12.348	12.250	*0.40%*	-	-	-
		Dry	18.767	34.048	*-28.93%*	-	-	-
		Season difference	*-20.63%*	*-47.08%*		-	-	-
	Individual fruit weight(kg)	Wet	0.084	0.084	*0.00%*	-	-	-
		Dry	0.103	0.104	*-0.69%*	-	-	-
		Season difference	*-10.06%*	*-10.75%*		-	-	-
Cucumis sativus	Plant productivity (kg)	Wet	1.258	1.451	*-7.13%*	-	-	-
		Dry	1.808	2.600	*-17.97%*	0.238	0.160	*19.57%*
		Season difference	*-17.93%*	*-28.36%*		-	-	-
	Fruit number	Wet	4.250	4.429	*-2.06%*	-	-	-
		Dry	6.563	7.846	*-8.91%*	1.333	3.167	*-40.74%*
		Season difference	*-21.39%*	*-27.84%*		-	-	-
	Individual fruit weight(kg)	Wet	0.293	0.335	*-6.68%*	0.178	0.052	*54.62%*
		Dry	0.274	0.333	*-9.68%*	-	-	-
		Season difference	*3.34%*	*0.32%*		-	-	-
Solanum lycopersicum	Plant productivity (kg)	Wet	1.752	1.556	*5.92%*	0.313	-	
		Dry	3.119	2.551	*10.02%*	0.465	0.330	*17.08%*
		Season difference	*-28.07%*	*-24.23%*		*-19.51%*	-	-
	Fruit number	Wet	9.030	3.013	*49.96%*	3.000	-	-
		Dry	23.413	23.640	*-0.48%*	4.500	7.500	*-25.00%*
		Season difference	*-44.33%*	*-77.39%*		*-20.00%*	-	-
	Individual fruit weight(kg)	Wet	0.811	0.989	*-9.88%*	0.103	-	-
		Dry	0.144	0.124	*7.47%*	0.098	0.044	*38.08%*
		Season difference	*69.91%*	*77.77%*		*2.48%*	-	-

General results	Season difference all crops			Treatment difference all crops			
	Productivity	Number of fruits	IFW	Season	Productivity	Number of fruits	IFW
	33%	*49%*	*12%*	Wet	*5%*	*13%*	*8%*
				Dry	*10%*	*17%*	*10%*

Table S2. Maximum plant height in cm reached by the studied species. Differences between treatments are expressed in percentage. Underlined values shows that control is higher than Groasis Waterboxx®.

	Capsicum	*Cucumis*		*Solanum*	
**Treatment/Island**	Santa Cruz	Santa Cruz	Floreana	Santa Cruz	Floreana
Groasis Waterboxx®	140.57	76.37	116.41	197.07	81.43
Control	159.04	157.30	95.00	243.10	55.02
Treatment Difference	*6.16%*	*34.63%*	*10.13%*	*10.46%*	*19.35%*

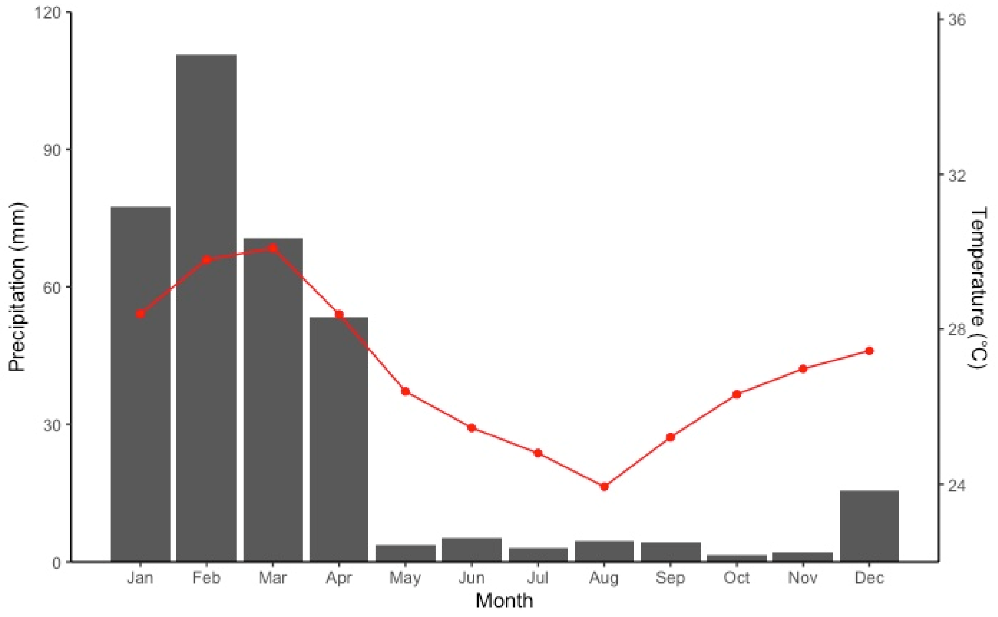

Figure S1.- Average monthly precipitation and temperature in the humid zone of Santa Cruz from 2016 to 2018. Average monthly precipitation is shown in gray and average temperature in red. Source: Galapagos Vital Signs: a satellite-based environmental monitoring system for the Galapagos Archipelago.

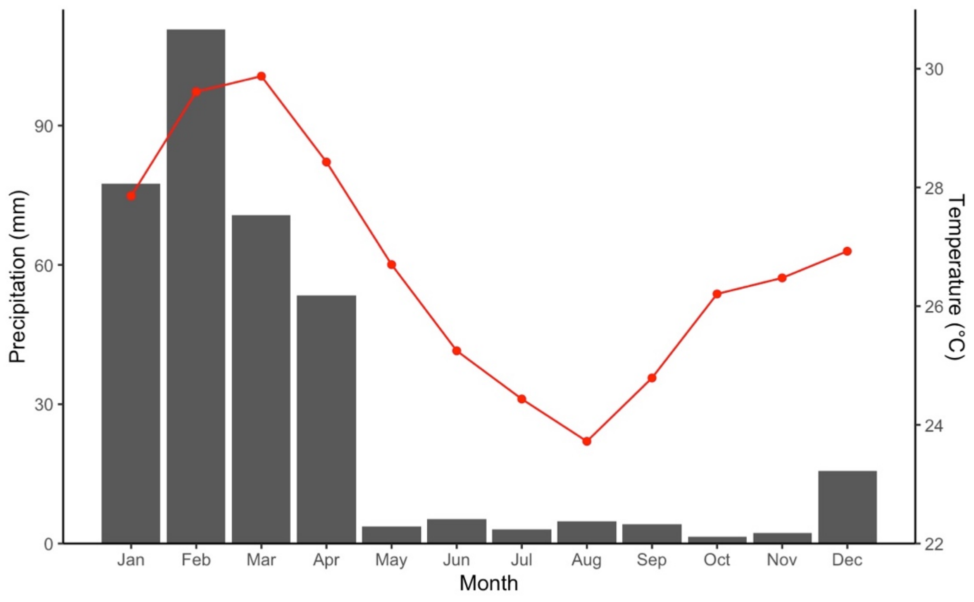

Figure S2.- Average monthly precipitation and temperature in Floreana from **2016 to 2018**. Average monthly precipitation is shown in gray and average temperature in red. Data source: Galapagos Vital Signs: a satellite-based environmental monitoring system for the Galapagos Archipelago

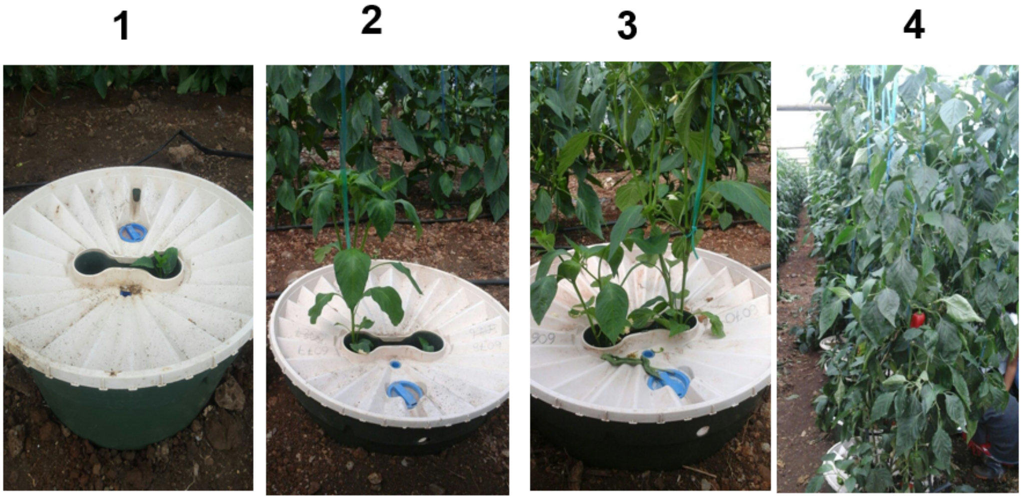

Figure S3.- Age classes that represent different stages for *Capsicum annuum*. Age class 1 represents (period or time registries) lectures from 1-17 days after transplanting DAT, age class 2 from 21-56 DAT, age class 3 from 63 to 98 DAT, and age class 4 from 112 to 204 DAT. Age classes represent: 1 early growth, 2 vegetative growth stage, 3) early flowering, and 4) harvest.

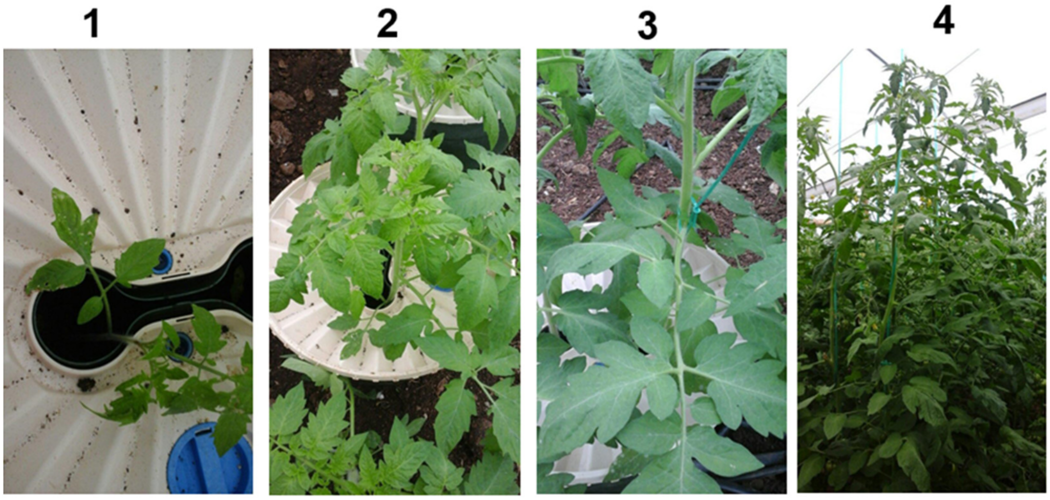

Figure S4.- Age classes that represent different stages for *Solanum lycopersicum*. Age class 1 represents lectures (period or time registries) from 1-10 days after transplanting DAT, age class 2 from 12-38 DAT, age class 3 from 44 to 84 DAT, and age class 4 from 88 to 130 DAT. Age classes represent: 1 early growth, 2 vegetative growth stage, 3) early flowering, and 4) harvest.
